# KI and WU Polyomaviruses and CD4+ Cell Counts in HIV-1–infected Patients, Italy

**DOI:** 10.3201/eid1609.100211

**Published:** 2010-09

**Authors:** Muhammed Babakir-Mina, Massimo Ciccozzi, Francesca Farchi, Massimiliano Bergallo, Rossana Cavallo, Gaspare Adorno, Carlo Federico Perno, Marco Ciotti

**Affiliations:** Author affiliations: Foundation University Hospital Tor Vergata, Rome, Italy (M. Babakir-Mina, G. Adorno, C.F. Perno, M. Ciotti);; Istituto Superiore di Sanita’, Rome (M. Ciccozzi, F. Farchi);; University of Turin, Turin, Italy (M. Bergallo, R. Cavallo)

**Keywords:** HIV-1, KI polyomavirus, WU polyomavirus, KIPyV, WUPyV, viruses, CD4+ cell counts, Italy, dispatch

## Abstract

To investigate an association between KI and WU polyomavirus (KIPyV and WUPyV) infections and CD4+ cell counts, we tested HIV-1–positive patients and blood donors. No association was found between cell counts and virus infections in HIV-1–positive patients. Frequency of KIPyV infection was similar for both groups. WUPyV was more frequent in HIV-1–positive patients.

BK and JC polyomaviruses are known to infect humans (*1**,**2*). Recently, the novel KI polyomavirus (KIPyV) and WU polyomavirus (WUPyV) have been identified in respiratory secretions of children with signs of acute respiratory disease (*3**,**4*). However, there is little evidence that these viruses are the causative agents of respiratory disease. The pathogenic role of these viruses in immunocompromised patients is also unclear.

In a study that investigated human polyomaviruses in autopsy lymphoid tissue samples from patients who were positive for HIV, KIPyV was detected in 7.1% of immunocompromised patients with AIDS and in 1.8% of nonimmunocompromised controls; WUPyV was detected in 9.5% of patients with AIDS but not in controls (*5*). We detected KIPyV and WUPyV in 3.2% and 1.6%, respectively, of plasma samples from HIV-1–infected patients (*6*). To determine an association between infection with KIPyV and WUPyV and CD4+ cell counts, we obtained plasma samples from HIV-1–positive patients having high and low CD4+ cell counts and a group of healthy controls and tested them for these 2 polyomaviruses.

## The Study

Plasma specimens from 153 HIV-1–infected persons (75% male patients, median age 41.9 years, interquartile range 33.8–47.3 years) with high (110 persons) and low (43 persons) CD4+ counts and from 130 blood donors (80% male donors, median age 41 years, interquartile range 32–47.5 years) were obtained at the Foundation Polyclinic Tor Vergata in Rome, Italy, during 2004–2009. Of 153 HIV-1–infected patients, 74 were receiving highly active antiretroviral therapy: a nucleoside reverse transcriptase inhibitor (NRTI) and a protease inhibitor (PI) (n = 35 patients); an integrase inhibitor (INI), an NRTI, and a PI (n = 7); a nonnucleoside-reverse transcriptase inhibitor and an NRTI (n = 26); an INI and an NRTI (n = 2); an INI and an NNRTI (n = 2); a chemokine receptor type 5 antagonist, an NRTI, and a PI (n = 1); and a chemokine receptor type 5 antagonist, an INI, and a nonnucleoside-reverse transcriptase inhibitor (n = 1). Sixty patients did not receive any therapy. No information was available for 19 patients. Additional information available for patients included HIV-1 viremia and co-infection with hepatitis B virus and hepatitis C virus.

Phylogenetic analysis of the small T antigen gene of KIPyV and WUPyV was performed as described (*6**,**7*). GenBank accession numbers of the sequences used in this analysis are shown in the Table A1.

Total DNA was extracted from 0.2-mL plasma samples by using QIAamp DNA Mini Kit (QIAGEN, Milan, Italy) according to the manufacturer’s instructions and stored at –80°C until analysis. Amplification of KIPyV and WUPyV was conducted as described (*8**,**9*). A standard curve was created in a 4-log range by using 1:10 serial dilutions of a virus-specific standard. The dynamic range was determined by using 10-fold dilutions (10^10^–100 copies/reaction) of each sample. Sensitivity of the 2 methods, which corresponded to the lowest plasma dilution detectable at a frequency of 100%, was evaluated. The dynamic range was 10^2^–10^10^ for KIPyV and 10^1^–10^10^ for WUPyV.

Statistical analysis was performed by using Epi Info version 3.5.1 software (Centers for Disease Control and Prevention, Atlanta, GA, USA). Odds ratios were determined for associations between infection with HIV and infection with KIPyV and WUPyV and other variables. Statistical significance was assessed by calculating 95% confidence intervals (CIs) and by using standard nonparametric statistics.

Real-time PCR detected KIPyV and WUPyV in 4 (2.6%) of 153 and 7 (4.6%) of 153 HIV-1–infected patients, respectively (Table 1). Of the 130 blood donors examined, 4 and 1 were positive for KIPyV (3.1%) and WUPyV (0.8%), respectively. For KIPyV, no difference was detected in the frequency of infection between HIV-1–infected patients and blood donors. Patients infected with HIV-1 had a higher risk for infection with WUPyV infection than did blood donors. However, this difference showed borderline statistical significance (odds ratio 6.15, 95% CI 0.93–141; p = 0.054). For WUPyV-positive and KIPyV-positive patients, median CD4+ cell counts were 308 cells/µL (95% CI 248–523 cells/µL) and 356 cells/µL (95% CI 270–517 cells/µL), respectively. No association was observed between CD4+ cell counts and risk for infection with KIPyV or WUPyV.

**Table 1:** Characteristics of 153 HIV+ persons tested for infection with WU and KI polyomaviruses, Italy, 2004–2009*†

Characteristic	WUPyV+, n = 7	WUPyV–, n = 146	KIPyV+, n = 4	KIPyV–, n = 149
CD4+ cells/µL				
<200	1 (14.3)	45 (30.8)		46 (30.9)
>200	6 (85.7)	101 (69.2)	4 (100)	103 (69.1)
Median (95% CI)	308 (248–523)	282 (153–378)	356 (270–517)	281 (154–378)
Virus load, copies/reaction				
<100,000	7 (100)	99 (67.8)	2 (50)	104 (69.8)
>100,000		47 (32.2)	2 (50)	45 (30.2)
Median (95% CI)	3,210 (108–36,895)	37,460 (5,490−152,000)	60,636 (2,791–246,500)	34,905 (4,980–145,200)
Co-infection				
Yes	2‡ (28.6)	29 (19.9)	2§ (50)	29 (19.5)
No	5 (71.4)	117 (80.1)	2 (50)	120 (80.5)

*Values are no. (%) unless otherwise indicated. WUPvV, WU polyomavirus; KIPyV, KI polyomavirus; CI, confidence interval. †Of the 153 persons tested, 115 (75.2%) were men, 38 (24.8%) women. A total of 130 blood donors (104 [80%] men, 26 [20%] women) were also tested; 1 (0.8%) was WUPyV+ and 4 (3.1%) were KIPyV+. ‡Co-infection with hepatitis virus. §Co-infection with hepatitis C virus and hepatitis B virus.

Median HIV-1 virus load in persons infected with WUPyV or KIPyV was 3,210 copies/mL (95% CI 108–36,895 copies/mL) or 60,636 copies/mL (95% CI 2,791–246,500 copies/mL), respectively. When HIV-1 virus load, CD4+ cell count, and co-infection with hepatitis B and C viruses were analyzed in patients infected with KIPyV or WUPyV, no association was found. Of 11 patients infected with KIPyV or WUPyV, 6 received highly active antiretroviral therapy and 5 did not receive any therapy (Table 2).

**Table 2:** Treatment regimens for 11 HIV-1–positive patients co-infected with KI or WU polyomavirus, Italy, 2004–2009*

Patient no.	KIPyV	WUPyV	HAART
1	+	–	None
2	+	–	None
3	+	–	None
4	+	–	NRTI: FTC, TDF; PI: ATV, RTV
5	–	+	NNRTI: EFV; NRTI: 3TC, TDF
6	–	+	NNRTI: NVP; NRTI: 3TC, AZT
7	–	+	NRTI: 3TC, AZT; PI: ATV, RTV
8	–	+	NNRTI: NVP; NRTI: ABC, TDF
9	–	+	None
10	–	+	NNRTI: EFV; NRTI: 3TC, D4T
11	–	+	None

*KIPyV, KI polyomavirus; WUPyV, WU polyomavirus; HAART, highly active antiretroviral therapy; NRTI, nucleoside reverse transcriptase inhibitor; FTC, emtricitabine; TDF, tenofovir; PI, protease inhibitor; ATV, atazanavir; RTV, ritonavir; NNRTI, nonnucleoside reverse transcriptase inhibitor; EFV, Efavirenz; 3TC, lamivudine; NVP, nevirapine; AZT, azidothymidine; ABC, D4T, stavudine.

Phylogenetic analysis showed that all WUPyVs identified in this study except WUV-IT4 are closely related to the WUV-IT3 strain identified in an HIV-1 patient (*6*) (Figure). The KIPyV strains identified are relatively distant from those identified in another study (*6*), except for strain KIV-RM23, which clusters with KIV-RM21 (*6*) (Figure).

**Figure:**
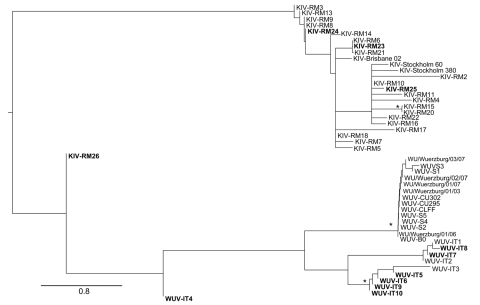
Maximum likelihood phylogenetic analysis of KI polyomavirus (KIPyV) and WU polyomavirus (WUPyV) small T antigen sequences. Strains identified in this study are in **boldface**. The tree was rooted by using the midpoint rooting method. Branch lengths were estimated by using the best fitting nucleotide substitution (Hasegawa, Kishino, and Yano) model according to a hierarchical likelihood ratio test (*6*,*7*) and were drawn to scale. Scale bar indicates 0.8 nt substitutions per site. Asterisks along the branches indicate significant statistical support for the clade subtending that branch (p<0.001 by the zero-branch-length test and bootstrap support >65%).

## Conclusions

KIPyV and WUPyV have been identified in respiratory secretions of pediatric patients (*3**,**4*). New polyomaviruses have also been detected in immunocompromised patients (*10**–**13*). However, the pathogenic role of these polyomaviruses in immunocompromised patients is unclear. No associations were found between CD4+ cell counts in HIV-1–positive patients and infection with KIPyV or WUPyV. Frequency of KIPyV infection for HIV-1–positive patients was similar to that for blood donors. However, frequency of WUPyV infection was higher for HIV-1–positive patients than for blood donors, although this difference showed borderline significance.

Detection of WUPyV did not show a correlation with virus load for HIV-1 or lower CD4+ cell counts. Seroprevalence of KIPyV and WUPyV in an adult population was 55% and 69%, respectively (*14*). The higher rate of infection for WUPyV may account for the higher rate of detection for WUPyV in our study population. In a previous study (*6*), prevalence of WUPyV in plasma of HIV-1–positive patients was lower than that in our study. This difference may have been caused by the larger sample size in our study.

Phylogenetic analysis did not suggest circulation of specific KIPyV and WUPyV strains in HIV-1–positive patients. The KIPyVs identified in this study cluster with those identified in HIV-1–positive patients, and the WUPyVs identified are closely related to the strain identified previously in an HIV-1–positive patient (*6*). However, these WUPyV strains also cluster with strain WUV-IT1 and 2 strains identified in stool samples (*10*).

Our study design and the complex nature of AIDS-related disease do not enable one to make definitive conclusions on the role of novel polyomaviruses in HIV-1–positive patients. However, our data seem to exclude an active role for KIPyV and WUPyV in HIV-1–positive patients.

We detected WUPyV and KIPyV in healthy persons and immunocompromised persons. BK and JC polyomaviruses persist in peripheral blood mononuclear cells in healthy persons (*15*). However, frequency of detection may vary from 0% to 90% of persons tested. This large variation may reflect recent infection or virus reactivation in a subgroup of persons (*15*). Thus, detection of KIPyV and WUPy in blood cells of immunocompetent persons is needed to identify a possible hematologic reservoir.

## Appendix

**Table A1:** KI and WU polyomavirus sequences used in phylogenetic analysis, Italy, 2004–2009

Virus	GenBank accession no.	Type of sample
KIV Stockholm60	NC_009238	Respiratory
KIV Brisbane 002	EF520288	Respiratory
KIV Stockholm 380	EF127908	Respiratory
WU/Wuerzburg/03/07	EU711058	Respiratory
WU/Wuerzburg/02/07	EU711057	Respiratory
WU/Wuerzburg/01/07	EU711056	Respiratory
WU/Wuerzburg/01/06	EU711055	Respiratory
WU/Wuerzburg/01/03	EU711054	Respiratory
WUV CU-302	EU358769	Respiratory
WUV CU-295	EU358768	Respiratory
WUV CLFF	EU296475	Respiratory
WUV S5	EF444554	Respiratory
WUV S4	EF444553	Respiratory
WUV S3	EF444552	Respiratory
WUV S2	EF444551	Respiratory
WUV S1	EF444550	Respiratory
WUV B0	EF444549	Respiratory
KIV-RM4	FJ389516	Lung tissue
KIV- RM3	FJ389515	Lung tissue
KIV- RM2	FJ389514	Lung tissue
KIV-RM5	FJ594126	Stool
KIV-RM6	FJ594124	Stool
KIV-RM7	FJ594125	Stool
KIV-RM8	FJ594120	Stool
KIV-RM9	FJ594121	Stool
KIV-RM10	FJ594122	Stool
KIV-RM11	FJ594123	Stool
WUVIT3	FJ842114	Plasma
KIV-RM22	FJ842113	Plasma
KIV-RM21	FJ842112	Plasma
WUV-IT2	FJ594119	Stool
WUV-IT1	FJ594118	Stool
KIV-RM13	FJ811519	Tonsil tissue
KIV-RM14	FJ811520	Tonsil tissue
KIV-RM15	FJ811521	Tonsil tissue
KIV-RM16	FJ811522	Tonsil tissue
KIV-RM17	FJ811523	Tonsil tissue
KIV-RM18	FJ811524	Tonsil tissue
KIV-RM20	FJ821706	Tonsil tissue
KIV-RM23	HM164689	Plasma
KIV-RM24	HM164690	Plasma
KIV-RM25	HM164691	Plasma
KIV-RM26	HM164692	Plasma
WUV-IT4	HM164682	Plasma
WUV-IT5	HM164683	Plasma
WUV-IT6	HM164684	Plasma
WUV-IT7	HM164685	Plasma
WUV-IT8	HM164686	Plasma
WUV-IT9	HM164687	Plasma
WUV-IT10	HM164688	Plasma
